# The Influence of the Phases of the Menstrual Cycle on Intrinsic Injury Risk Factors in Eumenorrheic Female Athletes or Physically Active Women—A Systematic Review

**DOI:** 10.3390/sports14070297

**Published:** 2026-07-10

**Authors:** Florent Osmani, Marta Victoria Santiago, Oliver Ramos-Álvarez

**Affiliations:** 1 Facultad en Ciencias de la Salud, Universidad Europea del Atlántico, 39011 Santander, Spain; florent.osmani@uneatlantico.es (F.O.); marta.santiago@uneatlantico.es (M.V.S.); 2 Research Group on Technology Applied to Research in Employment, Equality and Health (Talionis), University of A Coruña, 15403 A Coruña, Spain; 3 Health Economics and Health Services Management Research Group, Marqués de Valdecilla Research Institute (IDIVAL), 39011 Santander, Spain; 4 GESTAS Research Group, Exercise Physiology Research, Politécnico Colombiano Jaime Isaza Cadavid, Medellín 4412, Colombia

**Keywords:** sex hormones, oestrogens, progesterone, biomechanics, neuromuscular control, prevention, joint stability, sports medicine

## Abstract

Research in sports science has frequently excluded women due to the complexity of hormonal fluctuations. The overall aim of this study is to systematically analyse the influence of the menstrual cycle (MC) on intrinsic anatomical, biomechanical and neuromuscular injury risk factors in physically active women or eumenorrheic athletes. Methods: A systematic review was conducted following the guidelines of the PRISMA 2020 statement. Searches were carried out in PubMed, the Cochrane Library, Web of Science and Scopus up to March 2026, including studies that analysed at least three phases of the MC. Methodological quality was assessed using the Joanna Briggs Institute checklists. Results: Eleven articles were included in the analysis. It was observed that the late follicular and ovulatory phases were associated with improved biomechanical and neuromuscular profiles, thereby reducing certain risk factors. In contrast, the premenstrual and menstrual phases are associated with a more unfavourable effect, slightly increasing the risk of injury. Conclusion: Although phase-specific trends emerge, methodological heterogeneity and limited evidence preclude a definitive consensus on how the menstrual cycle impacts intrinsic injury risk factors. Nevertheless, practitioners should consider that late follicular and ovulatory phases present more favourable neuromuscular and biomechanical profiles than menstrual and premenstrual phases. Future research must utilize standardized phase verification to provide conclusive evidence.

## 1. Introduction

Women’s participation in sport has grown exponentially, with the number of female players in sports such as football tripling over the last decade [1]. Despite this boom, scientific research in sports science has always focused on men, excluding women due to the complexity of hormonal fluctuations [2]. There is a need for greater scientific focus on female physiology, given the many anatomical, biomechanical and endocrine differences that exist [2,3].

The menstrual cycle (MC) is a complex biological process regulated by the hypothalamic–pituitary–ovarian axis, which gives rise to periodic structural and functional changes [3]. A typical eumenorrheic menstrual cycle lasts between 21 and 35 days and is divided into the early and late follicular phases, ovulation, and the early, middle and late luteal phases [4]. Theoretically, a 28-day cycle is divided into four distinct phases based on fluctuations in oestradiol and progesterone: the early follicular phase (days 1–5), characterized by low concentrations of both hormones and the onset of menstruation; the late follicular phase (days 6–12), where oestrogen rises progressively whilst progesterone remains low; the ovulatory phase (days 13–15), marked by a peak and surge in luteinizing hormone (LH); and the luteal phase (days 16–28), which includes a mid-luteal window (days 20–23) where progesterone reaches its peak alongside a second rise in oestrogen [4,5,6].

From a biological perspective, it has been shown that sex hormones can influence musculoskeletal tissue. The anterior cruciate ligament (ACL) has receptors for oestradiol and progesterone, which may allow these hormones to alter collagen metabolism [3]. Specifically, high levels of oestrogen can reduce collagen density and increase joint laxity [7]. Furthermore, relaxing, a hormone that peaks during the luteal phase, may break down type 1 collagen, thereby compromising tissue quality and increasing the risk of mechanical failure under heavy loads [8].

The relationship between the phases of the menstrual cycle and the incidence of injuries is an area of study where there is no clear evidence. Whilst some classic studies link the ovulatory phase with an increased risk of ACL tears due to the peak in oestrogen levels, more recent research on elite athletes has observed a higher rate of muscle and tendon injuries during the luteal phase [9,10]. This variation in results is influenced by secondary factors such as perceived fatigue, poorer sleep quality and premenstrual symptoms, which affect more than 80% of female athletes and could increase the risk of injury [9,11]. This inconsistency in the data suggests that the risk of injury is multifactorial in nature and that there is no single, universally applicable phase of vulnerability [7,12].

Previous reviews are biased by earlier methodological issues, such as small sample sizes that lack statistical power, particularly as up to 40% of participants may require exclusion following rigorous verification [13,14]. Furthermore, phase identification is usually based on the calendar method, an imprecise estimation technique, given that theoretical 28-day menstrual cycles account for only 10 per cent of actual cases [13]. Lastly, there is a significant lack of hormonal verification; only 44% of studies measure actual oestrogen and progesterone concentrations [14]. Without biochemical confirmation, it is impossible to detect anovulatory or luteal phase-deficient cycles, which can affect up to 66% of exercising females [13].

Despite growing interest, no consensus exists regarding how MC phases influence intrinsic injury risk factors. So, the overall aim of this study is to systematically analyse the influence of the MC on intrinsic anatomical, biomechanical and neuromuscular injury risk factors in physically active women or eumenorrheic athletes.

## 2. Materials and Methods

### 2.1. Study Design and Registration

This systematic review has been conducted in accordance with the guidelines set out in the PRISMA 2020 statement [15]. It has been registered in Prospero under registration number CRD420261362817.

The databases used were: PubMed, the Cochrane Library, Web of Science and Scopus, with the search conducted in March 2026.

The search strategy was developed using a combination of keywords, namely: “menstrual cycle”, “estrogen”, “progesterone”, “follicular phase”, “luteal phase”, “menstruation, “ovulation”, “injury”.

The search query for the databases can be found in Supplementary Table S1.

### 2.2. Eligibility Criteria

The inclusion criteria were as follows: (i) women with a regular menstrual cycle (21–38 days); (ii) physically active women or athletes; (iii) testing carried out at least once during three different phases of the menstrual cycle; (iv) clear identification of the phases of the menstrual cycle during which testing was carried out; (v) relationship between the phases of the menstrual cycle and anatomical, biomechanical and neuromuscular intrinsic risk factors for sports injuries; (vi) intrinsic risk factors were operationally defined as any anatomical, biomechanical, or neuromuscular characteristics of the participant that could influence injury susceptibility; (vii) studies from January 2020 to April 2026; (vii) studies in English or Spanish.

The exclusion criteria were as follows: (i) women taking contraceptives; (ii) women who were injured at the time of the study; (iii) studies related to nutritional supplements; (iv) studies addressing extrinsic or intrinsic injury risk factors unrelated to sport.

### 2.3. Selection Process

The study selection and data collection processes were executed systematically by two independent reviewers (F.O. and O.R.A.), with a third author (M.V.S.) serving as an arbitrator. Initial literature screening was facilitated using the Rayyan software. Two reviewers independently screened the titles and abstracts of all retrieved records against the predefined inclusion and exclusion criteria. Discrepancies at this stage were resolved through consensus or, when necessary, by consulting the third author. The same independent, dual-review approach was applied to the full-text assessment of the remaining reports to determine final eligibility.

### 2.4. Data Extraction Process and Outcomes

**Figure 1:**
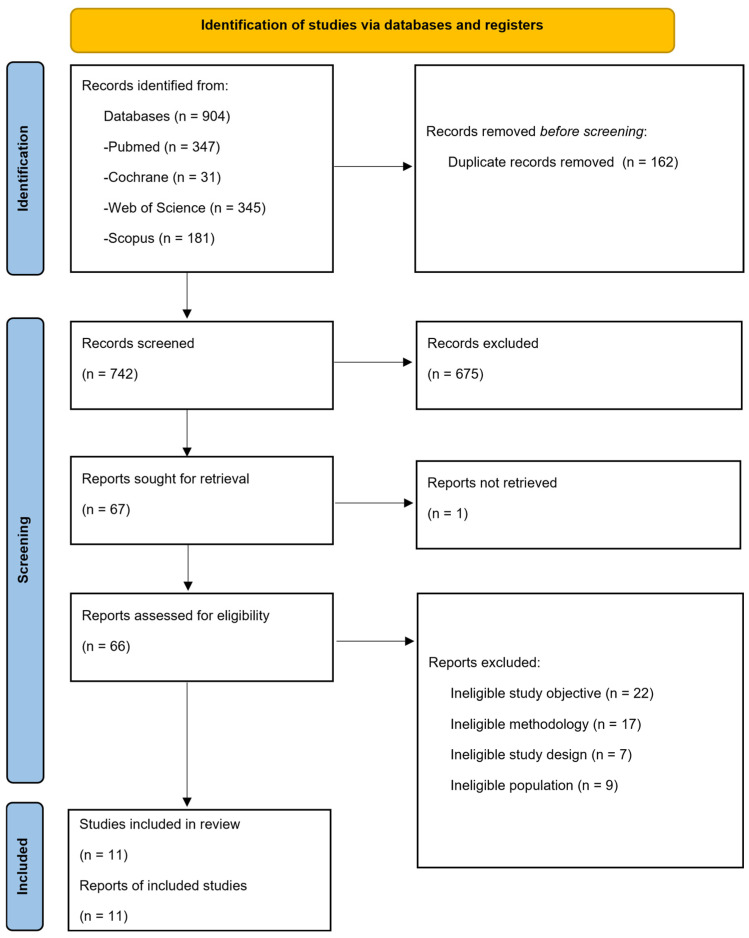
PRISMA flowchart. Adapted from the PRISMA 2020 statement.

Following study selection, data extraction was performed independently by the same two reviewers using a standardized data extraction form. The extracted information encompassed study characteristics, participant demographics, specific protocols for menstrual cycle phase verification, and intrinsic or extrinsic sports injury risk factors. Any disagreements during the data extraction phase were resolved via discussion between the two reviewers or through arbitration by the third author. Study investigators were not contacted for missing data, and no other automation tools were utilized beyond the initial screening software. A PRISMA flow diagram summarizes the selection process (Figure 1).

The primary outcomes sought for this review were intrinsic sports injury risk factors. Additionally, variables related to participant demographics, study characteristics, and specific protocols for menstrual cycle phase verification were extracted. This methodological classification of menstrual cycle verification (low, medium, or high quality) was utilized as a critical exposure variable to ensure that findings backed by biochemical and hormonal confirmation were assigned greater value during data synthesis.

### 2.5. Methodological Quality and Risk of Bias Assessment

The Joanna Briggs Institute (JBI) checklists were used to assess the methodological quality of the studies included in this review. These checklists enable an assessment of internal validity and risk of bias, differentiating criteria according to the specific design of each study. The checklists for cohort studies (11 items) were used [16] and for quasi-experimental studies (9 items) [17]. The scoring criteria for each item were: Yes, No, Not clear and Not Applicable.

Methodological quality and risk of bias of the included studies were assessed independently by two reviewers using the JBI checklists for cohort and quasi-experimental studies. Any discrepancies or disagreements during this evaluation were resolved through discussion between the two reviewers or via arbitration by the third author. No automation tools or specialized software were utilized during the risk of bias assessment process.

### 2.6. Menstrual Cycle Phase Verification Quality Assessment

Given the critical importance of methodological accuracy highlighted in the introduction, a specific assessment was carried out of the quality of menstrual cycle verification, classifying the studies according to the criteria proposed in the recent literature [4,6]:

− Low quality: Verification based solely on the calendar method or memory.
− Medium quality: A combination of the calendar method with basal body temperature or an LH ovulation test kit.
− High quality: Biochemical confirmation of hormonal peaks via blood tests.

### 2.7. Data Synthesis and Analysis

Due to the substantial heterogeneity in study designs, specific sports tasks, and measured biomechanical and clinical outcome parameters, a quantitative meta-analysis was not feasible. Instead, a narrative and descriptive synthesis approach was implemented. Studies were grouped and compared qualitatively based on participant characteristics and the specific category of intrinsic injury risk factor investigated, such as joint laxity, kinematics, range of motion, and muscular torque or strength. Data were systematically compiled and visually presented using a structured table detailing the author, study aim, effective sample size, analysed variables, investigated menstrual cycle phases, and primary findings. No data conversions or statistical transformations were performed to handle missing data or summary statistics. Methodological quality scores were not used as a restrictive exclusion criterion, and a formal sensitivity analysis was not performed due to data heterogeneity. Instead, risk of bias was considered when interpreting results; low-quality studies or those lacking objective tracking were down-weighted during data synthesis, meaning that their findings contributed less to establishing final trends. Consequently, studies with high-quality hormonal verification were prioritized in interpreting findings and drawing conclusions.

## 3. Results

### 3.1. PRISMA Flowchart

Figure 1 shows the PRISMA flow chart, which began with 904 articles, with 11 articles remaining for analysis following the complete screening process. A total of 66 full-text articles were assessed for eligibility, of which 55 were excluded after rigorous evaluation (see Supplementary Table S2 for the full list of excluded studies and reasons). Finally, 11 studies met all criteria and were included in the review.

### 3.2. Study Characteristics

Table 1 lists the included studies, which examine the relationship between the phases of the MC and intrinsic injury risk factors. In total, 11 studies with a total of 235 athletes with eumenorrheic MC were included in the review. These studies have been analysed with a focus on populations such as: physically active women (9.1%) [18], athletes (9.1%) [19], team sports players (45.5%) [9,20,21,22,23], high-level (18.2%) [24,25] or high risk of injury to the anterior cruciate ligament (9.1%) [26], or overhead sports (9.1%) [27] (Table 1).

### 3.3. Synthesis of Results

Regarding joint laxity and passive mechanical properties, Maruyama et al. [21] reported significant fluctuations in passive joint laxity, noting that genu recurvatum increased significantly during the late follicular (*p* = 0.050), ovulatory (*p* = 0.011), and luteal (*p* = 0.004) phases compared to the early follicular phase, alongside slightly higher general joint laxity during the ovulatory and luteal phases. Conversely, Bouvier et al. [18] found no statistically significant differences in passive hamstring stiffness, elasticity, maximum voluntary contraction, or muscle anatomical cross-sectional area across the evaluated MC phases.

In terms of kinematics and movement dynamics, Bingzheng et al. [22] observed a significantly lower maximum knee valgus during 90° changes of direction in the mid-luteal phase (4.9°) compared to the other phases(6.6–6.7°; *p* < 0.05). Domínguez-Muñoz et al. [19] identified isolated kinematic alterations during running, demonstrating a significantly lower vertical velocity (*p* = 0.004 vs. early follicular phase; *p* = 0.003 vs. mid-luteal phase) and a higher stride frequency (*p* = 0.017 vs. mid-luteal phase) exclusively during the late follicular phase. Meanwhile, Sajjadi et al. [24] found no significant differences (*p* > 0.05) in hip, knee, or ankle joint ranges of motion, nor in inter-joint coordination variability during taekwondo roundhouse kicks across the early follicular, ovulatory, and mid-luteal phases.

Evaluating muscular strength and torque production, Pournasiri et al. [26] and Forouzandeh Shahraki et al. [27] both reported that peak muscle strength was in the same line during the ovulatory phase, with Pournasiri et al. [26] demonstrating significantly higher isometric and isokinetic strength in both knee extensors and flexors (*p* < 0.001) and Forouzandeh Shahraki et al. [27] reporting significantly better shoulder abductor and internal/external rotator strength (*p* < 0.05). Additionally, Johnson et al. [25] determined that knee extension eccentric torque in elite athletes was significantly higher in the mid-luteal phase than in the late follicular phase (*p* < 0.05).

Concerning neuromuscular control, stability, and proprioception, Quigley & Greig [23] showed that the functional range of eccentric strength in the knee flexors decreased significantly during the early follicular phase (16.36°) compared to ovulation (21.15°) and the mid-luteal phase (20.69°; *p* < 0.05). Furthermore, Kacem et al. [20] demonstrated that a fatigue protocol significantly impaired postural control, reduced dynamic balance reach, and increased centre of mass oscillation area to a greater extent during the premenstrual phase compared to the very late follicular and mid-luteal phases (*p* < 0.001). This matches the findings of Forouzandeh Shahraki et al. [27], who documented significant proprioceptive deficits and greater joint repositioning errors in the shoulder during the mid-luteal phase when compared directly to the ovulatory phase (*p* < 0.05).

Regarding the epidemiological injury incidence, Fort-Vanmeerhaeghe et al. [9] reported that 78.4% of total recorded injuries occurred during the early and late luteal phases (*p* = 0.012), with joint, ligament, tendon, and myotendinous injuries being the most prevalent. This statistical concentration of injuries coincided with significantly higher levels of perceived fatigue and poorer sleep quality reported by the athletes during these same luteal phases (*p* < 0.001).

### 3.4. Methodological Quality

Table 2 presents the quality assessment of quasi-experimental and cohort studies respectively. The Joanna Briggs Institute’s checklists were used for this purpose [16,17]. This table presents an analysis of the cause-and-effect relationship, the heterogeneity of the participants, whether the same treatment was used, whether there was a control group, whether repeated measurements were taken, whether there was a follow-up, whether the measurement was the same for everyone, whether the measurement was reliable, and whether the statistical method was appropriate.

Table 3 details the quality assessment of the cohort studies based on the JBI checklist. In this instance, the analysis examined whether the groups were comparable, whether participants’ exposure was consistent and valid, whether there were confounders or confounding strategies, whether the results were valid, whether the study duration was sufficient and complete with no data loss, and whether the statistical analysis was appropriate.

Table 4 shows the quality assessment of the studies based on the determination of the MC phases:

1. Low quality: Verification based solely on the calendar or recall method.
2. Medium quality: Combination of the calendar method with basal body temperature or symptom tracking.
3. High quality: Biochemical confirmation of hormonal peaks (oestrogen, progesterone, and LH) via blood, urine or saliva analysis.

Regarding methodological quality, the included studies generally exhibited a low risk of bias according to the JBI criteria, with cohort studies scoring between 88.8% and 100% and quasi-experimental studies ranging from 66.6% to 83.3%. These high scores indicate that both cohort and quasi-experimental designs possess robust structural internal validity, consistent exposure measurements, and appropriate statistical frameworks to track chronological variations, making them structurally sound. However, the interpretation of these quality scores changes substantially when accounting for MC verification precision (Table 4). Only 3 out of 11 studies (27.3%) were classified as high quality due to rigorous biochemical confirmation via blood testing, which successfully minimizes phase-misclassification bias. In contrast, four studies (36.4%) presented moderate methodological quality by combining calendar tracking with LH kits or temperature monitoring, and the remaining four studies (36.4%) fell into the low-quality category due to reliance on the calendar method or self-reporting alone. This indicates that while internal study structures are robust, over a third of the current literature carries a significant risk of biological phase-misclassification bias due to the potential inclusion of undetected anovulatory or luteal-phase-deficient cycles, meaning that their physiological findings must be interpreted with caution.

## 4. Discussion

### 4.1. Competing Effects: Structural Vulnerability vs. Neuromuscular Compensation

The main findings of the 11 studies included in this systematic review regarding the influence of the MC phases on intrinsic injury risk factors in eumenorrheic women, both physically active individuals and athletes, do not reveal a conclusive pattern. Rather than a direct relationship, our synthesis suggests that MC phases produce competing physiological effects. We propose that structural vulnerability may coexist with neuromuscular compensation, resulting in inconsistent net risk profiles across the cycle. On the one hand, the results of our review indicate that the late follicular and ovulatory phases are associated with a reduction in the prevalence of certain risk factors, manifesting more favourable biomechanical and neuromuscular profiles in running kinematics [19], muscle stiffness [19], isometric and isokinetic flexion-extension strength of the knee [23,26], the abductor and rotator muscles of the shoulder [27] and the range of motion in ankle dorsiflexion [24]. In contrast, the premenstrual and menstrual phases appear to have an adverse effect on these intrinsic parameters, potentially increasing susceptibility to injury [9,19,20,21,23,26,27]. Specifically, the mid-luteal phase demonstrates a more complex profile; while structural vulnerability may increase, our findings suggest it coexists with protective neuromuscular adaptations, such as increased eccentric torque that may mitigate the overall risk of injury [21,25]. However, the current evidence is contradictory; these discrepancies highlight the multifactorial nature of injury risk and appear to be influenced by the diversity of the markers analysed, inter-individual hormonal fluctuations, and variations in the methodological rigour of existing studies.

Analysis of intrinsic risk factors reveals a clear contradiction between passive structural integrity and dynamic technical execution. On the one hand, an increase in passive joint instability is observed during phases of high hormonal activity. Maruyama et al. [21] demonstrated that genu recurvatum increases significantly during the late follicular, ovulatory and luteal phases compared with the early follicular phase. This increase in laxity could reduce the ligaments’ ability to withstand mechanical loads, as elevated oestrogen levels may decrease collagen density. Supporting this, Balachandar et al. [28] report that the highest risk of ACL injury occurs during the pre-ovulatory phase, when peak oestrogen levels inhibit type I collagen synthesis and fibroblast proliferation. This biochemical weakening, combined with increased knee valgus and tibial external rotation, significantly compromises the ligament’s structural integrity and its ability to withstand mechanical loads.

However, the results of our analysis suggest that this structural vulnerability coexists with dynamic neuromuscular compensation, indicating that the phases of the MC produce opposing effects rather than a linear relationship. This capacity for functional adaptation is supported by findings from high-quality designs in our synthesis showing protective movement patterns. So Bingzheng et al. [22], employing a high-quality study design with biochemical confirmation, observed a lower maximum knee valgus (4.9°) during the mid-luteal phase compared with other phases such as the late follicular or ovulatory phases (6.6–6.7°). This suggests that female athletes adopt protective movement patterns, implying active compensation by the neuromuscular system in response to ligamentous laxity. Furthermore, the evidence of a protective window in the second half of the cycle is reinforced by Johnson et al. [25], who found that the eccentric torque of the knee extensors is 11% higher during the mid-luteal phase compared with the late follicular phase.

This capacity for functional adaptation is supported by the findings of Bouvier et al. [18], one of the high-quality studies included in our synthesis, which reported no significant changes in hamstring stiffness or cross-sectional area throughout the cycle. This finding challenges the traditional view of uniform laxity throughout the musculoskeletal system, suggesting that certain muscle properties remain relatively stable in physically active women. The systematic review by Dos’Santos et al. [12] notes that the evidence remains inconclusive as to whether a specific phase of the MC predisposes women to a higher biomechanical risk, due to the high individual variability in response to fluctuations in oestradiol and progesterone.

### 4.2. Muscular Strength and Functional Control

Meanwhile, the ability to generate force is a critical factor in joint stabilization and injury prevention, particularly during deceleration and changes of direction. The results of this review point to ovulation as the point of peak strength performance, acting as a primary example of how neuromuscular compensation can provide a window of protection. Although it should be noted that in certain studies the ovulatory phase may still be considered part of the late follicular phase, this depends on the methodological rigour of each study. In research such as that by Pournasiri et al. [26] and Forouzandeh Shahraki et al. [27], it is agreed that peak strength, both isokinetic and isometric, in the knee and shoulder reaches its highest levels during the ovulatory phase, likely driven by the peak in oestradiol. This increase in strength could act as a temporary protective mechanism; however, this advantage disappears during phases of low hormonal availability (menstrual phase) or in situations of accumulated fatigue, where the risk of injury increases considerably.

A finding of particular relevance for prevention is the reduction in the functional range of eccentric strength described by Quigley & Greig [23]. This concept refers to the ability to maintain high levels of force at specific joint angles, beyond the absolute maximum value. The authors observed that the ability of the knee flexors to sustain levels above 85% of maximum force decreases significantly during the early follicular or menstrual phase (16.36° compared with 21.15° at ovulation). This limits neuromuscular control at critical joint positions and increases the likelihood of mechanical failure during demanding tasks.

The results compiled in our systematic review are consistent with the meta-analysis by Niering et al. [29], which identifies the early follicular phase as the least favourable period for maximum strength performance, whilst the late follicular phase is the most favourable for maximum isometric and dynamic strength, albeit with a moderate and slight effect respectively.

In terms of sport-specificity and biomechanical response, it is observed that the late follicular and ovulatory phases may offer certain advantages or protective adaptations. In cyclic and high-impact tasks such as running, Domínguez-Muñoz et al. [19] observed lower vertical velocity and a higher stride frequency during the late follicular phase. This change in shock-absorption mechanics should be analysed in conjunction with the viscoelastic properties of the muscle; indeed, Maruyama et al. [21] suggest that changes in stiffness during these phases of high oestrogen levels could contribute to a more efficient biomechanical profile.

### 4.3. Epidemiological Impact and the Role of Fatigue

This window of improvement, which culminates in the ovulatory phase, is also reflected in the execution of highly specialized technical movements. Sajjadi et al. [24], when analysing the roundhouse kick in taekwondo, found no impairment in inter-joint coordination patterns or ROM during ovulation, suggesting that the ovulatory hormonal peak might facilitate, or at least not hinder, advanced motor control and technical mastery.

As for the incidence of injuries, it appears to be critically concentrated in the second half of the cycle, suggesting the existence of a window of increased vulnerability associated with the luteal hormonal environment. Fort-Vanmeerhaeghe et al. [9] reported a striking epidemiological finding: 78.4% of injuries recorded in elite adolescents occurred during the early and late luteal phases. This increase in injury rates is not due to a single factor but rather to a “risk triangle” comprising fatigue, poorer sleep and proprioceptive deficits. The athletes themselves reported poorer sleep quality and greater fatigue during this luteal phase, which could impair their ability to recover [9].

From a neuromuscular perspective, the evidence supports this model. Kacem et al. [20] demonstrated that the impact of fatigue on postural control and dynamic balance is significantly greater during the premenstrual phase compared with the follicular and mid-luteal phases. This suggests that the neuromuscular system has a reduced capacity to compensate for external disturbances during this period. Similarly, Forouzandeh Shahraki et al. [27] observed a significant increase in joint repositioning error during the mid-luteal phase compared with the ovulatory phase, indicating a reduction in proprioceptive accuracy.

### 4.4. Methodological Considerations and Future Directions

A key aspect of this review is the analysis of methodological quality, as the reliability of the conclusions depends directly on the accuracy of the MC verification. Of the 11 studies included, only 27.3% [18,22,25] used biochemical confirmation of hormonal peaks, considered the “gold standard” in women’s sports research. The remaining studies based their measurements on less accurate methods, such as the calendar method or self-reporting, which increases the risk of including anovulatory cycles or those with luteal insufficiency, thereby masking potential real hormonal effects.

This level of evidence is consistent with that reported in the high-impact literature. The review by Dos’Santos et al. [12] notes that the quality of the evidence regarding risk factors for ACL injury during the menstrual cycle is generally “low to very low”, which means that the findings are often inconclusive. Similarly, the meta-analysis by McNulty et al. [4] warns that, although a slight reduction in response is observed in the early follicular phase, the considerable variability between studies means that the data must be interpreted with caution and that an individualized approach should be prioritized. It is imperative that future research follows the consensus guidelines set out by Elliott-Sale et al. [6] by incorporating analyses of hormones in blood or saliva and broader samples to translate these trends into definitive clinical recommendations.

### 4.5. Limitations

This review has several limitations that should be taken into account when interpreting the results. Firstly, there is considerable methodological heterogeneity among the included studies, particularly regarding the verification of menstrual cycle phases. Only a minority employed biochemical confirmation of hormonal profiles, considered the “gold standard”, whilst the majority used indirect methods such as the calendar method or self-reporting, which increases the risk of misclassification of phases and may mask actual hormonal effects.

Secondly, variability in sample characteristics, sporting level, type of discipline and the variables analysed limits comparability between studies and the generalization of results. Furthermore, the small sample size in several studies may have compromised statistical power.

Thirdly, the total number of included studies is relatively small (*n* = 11), which restricts the ability to generalize these trends broadly across different sporting populations.

Furthermore, the multifactorial nature of injury risk poses an inherent limitation, as factors such as fatigue, sleep, training load or individual biomechanical characteristics are not consistently controlled for and may act as confounding variables.

Also, there is a pronounced lack of standardized outcome measures across the literature, as studies evaluated highly diverse biomechanical, clinical, and neuromuscular variables, preventing a cohesive quantitative synthesis.

Finally, the predominance of observational or quasi-experimental designs prevents the establishment of causal relationships, so the findings should be interpreted with caution.

## 5. Conclusions

Current scientific evidence presents inconclusive results regarding the influence of the menstrual cycle phases on intrinsic injury risk factors. Whilst there appears to be a trend towards a reduction in these factors during the late follicular and ovulatory phases, in contrast to a potential increase in vulnerability during the premenstrual and menstrual phases, there is insufficient evidence to make definitive claims. Consequently, further research with more robust methodological designs is required, particularly regarding the standardization and clinical verification of the menstrual cycle phases. This will enable precise comparisons to be made and a more rigorous analysis of how underlying hormonal fluctuations actually modulate the risk of injury in the female population.

From a practical standpoint, MC phases must be integrated into training periodization due to phase-specific variations in injury risk factors, which may be exacerbated by high training loads or intensities during vulnerable periods. Furthermore, responses to hormonal fluctuations exhibit significant inter-individual variability; therefore, athlete monitoring should be highly individualized to avoid misleading generalizations when translating group data to practical environments.

## Figures and Tables

**Table 1:** Characteristics of studies examining the relationship between the phases of the menstrual cycle and intrinsic risk factors.

Author and Year	Aim of the Study	Sample (Total/ Effective Sample)	Variables	MC Phases Analysed	Key Findings
Bingzheng et al. (2023) [22]	Effect of the menstrual cycle phases on knee kinematics during changes of direction	31 out of 31 female university footballers	Maximum knee valgus during 90° changes of direction	Menstrual, late follicular, ovulatory and mid-luteal phases	Lower maximum knee valgus in the mid-luteal phase (4.9°) compared to other phases (6.6–6.7°), indicating a lower biomechanical risk of injury (*p* < 0.05).
Bouvier et al. (2025) [18]	Relationship between CM phases and passive mechanical properties of the hamstrings	40/20 active women who exercise regularly; MC: 30.4 days	Muscle stiffness, anatomical cross-sectional area of the hamstrings, elasticity and maximum range of motion	Early follicular, late/ovulatory (day 16.2) and mid-luteal phases	No significant differences were found in any of the variables relating to stiffness, maximum voluntary contraction, electromyographic activity or anatomical cross-sectional area of the muscle.
Domínguez-Muñoz et al. (2024) [19]	Changes in running kinematics and intensity according to menstrual cycle phases	8 out of 8 women with 3 years’ running experience + 3 h/week of regular running (24–38 days)	Stride length and angle, stride frequency, vertical velocity, ground contact and flight times, maximum pronation and stance velocities	Early follicular, late follicular and mid-luteal phases	Most running kinematic variables show no significant difference. Lower vertical velocity in the late follicular phase (*p* = 0.004 vs. early follicular phase (EFP) and 0.003 vs. mid-luteal phase (MLP)), higher stride frequency (*p* = 0.017 vs. MLP)
Forouzandeh Shahraki et al. (2020) [27]	Changes in shoulder stability factors during the phases of the menstrual cycle	15 out of 15 athletes performing overhead movements with a menstrual cycle of 26–32 days	Strength (abduction, internal/external rotation), proprioception (sense of joint position), ligament laxity and functional stability (upper limb Y balance test (YBT))	Menstruation (day 4), ovulation (24–48 h post-ovulation test) and mid-luteal phase (7 days post-positive ovulation)	Significant differences (*p* < 0.05) in abductor strength, internal and external shoulder rotation, with better performance in the ovulatory phase, and in proprioception, with poorer performance in the mid-luteal phase compared to the ovulatory phase.
Fort-Vanmeerhaeghe et al. (2025) [9]	Questionnaire on well-being and injuries over a 6-month period in relation to the phases of the menstrual cycle	59/59 elite team athletes; mean age: 28 ± 7	Incidence of injuries (type, severity, location), sleep quality, perceived fatigue, stress and musculoskeletal pain	Early/late follicular and early/late luteal phases	In total, 78.4% of injuries occurred in the early and late luteal phases (*p* = 0.012), with joint, ligament, tendon and myotendinous injuries being the most common during these phases. Poorer sleep quality and greater fatigue were observed in the early and late luteal phases (*p* < 0.001).
Johnson et al. (2026) [25]	Impact of the phases of the menstrual cycle on eccentric torque of the knee extensors	21/17 elite athletes with a menstrual cycle of 21–35 days	Eccentric torque of knee extension and isometric torque of knee extension and flexion	Early follicular (days 1–5), late follicular (high oestrogen levels) and mid-luteal (7–9 days after the LH peak)	Eccentric torque of the knee extensors was significantly higher in the mid-luteal phase than in the late follicular phase (*p* < 0.05).
Kacem et al. (2021) [20]	Effect of fatigue on postural control according to the phases of the menstrual cycle	15/12 handball players, 11 h of weekly training; 28-day menstrual cycle	Centre of mass oscillation area, centre of mass length (medio-lateral and antero-posterior) and Y-balance test (YBT)	Very late follicular (day 13), mid-luteal and premenstrual (day 27)	Fatigue significantly impaired postural control and reduced maximum voluntary isometric strength and reach in the Y-balance test in the premenstrual phase compared with the other phases (*p* < 0.001).
Maruyama et al. (2022) [21]	Knee laxity, stiffness and genu recurvatum (GR) (knee extension) during the different phases of the menstrual cycle	34/8 female university athletes (volleyball and basketball)	Anterior knee laxity, knee stiffness, general joint laxity and genu recurvatum (knee extension)	Early/late follicular, ovulatory and luteal phases	Genu recurvatum was significantly higher in the late follicular, ovulatory and luteal phases compared to the early follicular phase (*p* = 0.050, 0.011, 0.004 respectively). Stiffness was slightly higher in the late follicular and luteal phases, whilst joint laxity was slightly higher in the ovulatory and luteal phases, although knee laxity was lower in the luteal phase.
Pournasiri et al. (2023) [26]	Isometric/isokinetic strength of knee extensors and flexors according to phases of the menstrual cycle	37 out of 37 athletes participating in sports with a high risk of ACL injury; menstrual cycle duration: 21–35 days	Isometric and isokinetic strength of the knee extensors and flexors	Follicular (days 1–9), ovulatory (10–14) and luteal (15–28)	Greater isokinetic and isometric strength of knee extensors and flexors in the ovulatory phase compared with the follicular and luteal phases (*p* < 0.001).
Quigley & Greig (2025) [23]	The influence of the menstrual cycle phases on isokinetic knee strength in female footballers	8 out of 8 female university footballers	Maximum torque, angle of maximum torque and functional range (the angular range within which ≥85% of maximum torque is maintained) of the knee	Early follicular phase (day 2), ovulation (day 14) and luteal phase (day 21)	The functional range of eccentric strength in the knee flexors decreased significantly in the early follicular phase (16.36° compared to 21.15° at ovulation and 20.69° in the mid-luteal phase; *p* < 0.05).
Sajjadi et al. (2025) [24]	Effect of the phases of the menstrual cycle on the kinematics of taekwondo kicks	20 professional taekwondo athletes; menstrual cycle duration of 28–31 days	Range of joint motion (hip, knee, ankle) and variability in inter-joint coordination during taekwondo kicks	Early follicular, ovulation (13–15 days) and mid-luteal	No significant differences were found in the range of motion of the ankle, knee and hip between phases of the menstrual cycle (*p* > 0.05). A slight increase in the range of motion (ROM) of knee flexion-extension and ankle plantar flexion and dorsiflexion was observed in the ovulatory phase.

Note. Compiled by the authors. MC: menstrual cycle; ACL: anterior cruciate ligament; EFP: early follicular phase; MLP: mid-luteal phase; ROM: range of motion.

**Table 2:** Quasi-experimental studies.

Estudy	Q1	Q2	Q3	Q4	Q5	Q6	Q7	Q8	Q9	Score (%)
Bingzheng et al., 2023 [22]	Y	NA	NA	N	Y	Y	NA	Y	Y	83.3%
Domínguez-Muñoz, 2024 [19]	Y	NA	NA	N	Y	Y	NA	Y	Y	83.3%
Forouzandeh Shahraki et al., 2020 [27]	Y	NA	NA	N	Y	Y	NA	Y	Y	83.3%
Johnson et al., 2026 [25]	Y	NA	NA	N	Y	N	NA	Y	Y	66.6%
Kacem et al., 2021 [20]	Y	NA	NA	N	Y	N	NA	Y	Y	66.7%
Pournasiri et al., 2023 [26]	Y	NA	NA	N	Y	Y	NA	Y	Y	83.3%
Quigley & Greig, 2025 [23]	Y	NA	NA	N	Y	Y	NA	Y	Y	83.3%
Sajjadi et al., 2025 [24]	Y	NA	NA	N	Y	Y	NA	Y	Y	83.3%

Note. JBI Quasi-experimental items: Q1: clear cause-and-effect relationship; Q2: similar participants; Q3: similar treatment; Q4: control group?; Q5: repeated measurements; Q6: follow-up; Q7: consistent measurement; Q8: reliable measurement; Q9: statistics. Key: Y = Yes; NA = Not Applicable; N = No.

**Table 3:** Cohort studies.

Estudy	Q1	Q2	Q3	Q4	Q5	Q6	Q7	Q8	Q9	Q10	Q11	Score
Bouvier et al., 2025 [18]	Y	Y	Y	Y	Y	NA	Y	Y	Y	NA	Y	100%
Fort-Vanmeerhaeghe, 2025 [9]	NA	Y	N	Y	Y	Y	Y	Y	Y	NA	Y	88.8%
Maruyama et al., 2022 [21]	Y	Y	Y	Y	Y	NA	Y	Y	Y	NA	Y	100%

Note. JBI cohort items: Q1: similar groups; Q2: equal exposure; Q3: valid exposure; Q4: confounders; Q5: confounder strategy; Q6: free from baseline outcome; Q7: valid outcomes; Q8: sufficient time; Q9: complete follow-up; Q10: loss to follow-up; Q11: statistics. Key. Y = Yes; NA = Not Applicable; N = No.

**Table 4:** Quality assessment of the determination of MC phases.

Author and Year	Calendar Method or Self-Monitoring	Combination of the Calendar Method with Basal Body Temperature or LH Confirmation via Urine Test	Biochemical Confirmation of Hormone Surges via Blood Test	Final Result
Bingzheng et al., 2023 [22]			X	High
Bouvier et al., 2025 [18]			X	High
Domínguez-Muñoz et al., 2024 [19]	X (It does not specify exactly)			Low
Forouzandeh Shahraki et al., 2020 [27]		X		Medium
Fort-Vanmeerhaeghe et al., 2025 [9]	X			Low
Johnson et al., 2026 [25]			X	High
Kacem et al., 2021 [20]		X		Medium
Maruyama et al., 2022 [21]		X		Medium
Pournasiri et al., 2023 [26]	X			Low
Quigley & Greig, 2025 [23]		X		Medium
Sajjadi et al., 2025 [24]	X			Low

Note. Own work.

## Data Availability

All data supporting the findings of this study are qualitatively synthesized and fully presented within this published article and its accompanying summary tables. The extraction forms used during the review process are available from the corresponding author upon reasonable request.

## Supplementary Materials

The following supporting information can be downloaded at: https://www.mdpi.com/article/10.3390/sports14070297/s1, PRISMA Checklist; Table S1: Search strategy for each database; Table S2: Studies excluded after full text reading with the reason for exclusion.

## Abbreviations

The following abbreviations are used in this manuscript:

ACL	Anterior Cruciate Ligament
EFP	Early Follicular Phase
JBI	Joanna Briggs Institute
LH	Luteinizing Hormone
MC	Menstrual Cycle
MLP	Mid-Luteal Phase
ROM	Range of Motion
YBT	Y Balance Test
