# Supplementary material for: The Influence of the Phases of the Menstrual Cycle on Intrinsic Injury Risk Factors in Eumenorrheic Female Athletes or Physically Active Women—A Systematic Review
Source: Sports (Basel). 2026 Jul 10;14(7):297. doi: 10.3390/sports14070297 (PMC13416997; doi:10.3390/sports14070297)
Supplement: Supplementary file 1 [file sports-14-00297-s001.zip › sports-4372148-Table S1.pdf]

Supplementary Table S1.

Search strategy for each database.

**PubMed**
((((((("menstrual cycle"[Title/Abstract]) AND (estrogen[Title/Abstract])) OR (progesterone[Title/Abstract])) OR ("follicular phase"[Title/Abstract])) OR ("luteal phase"[Title/Abstract])) OR (menstruation[Title/Abstract])) OR (ovulation[Title/Abstract])) AND (injury[Title/Abstract])) NOT (review[Publication Type])
**Scopus**
"menstrual cycle" (Topic) AND estrogen OR progesterone OR "follicular phase" OR "luteal phase" OR menstruation OR ovulation (Topic) AND injury (Topic) and Review Article (Exclude – Document Types) and English or Spanish (Languages) and 2026 or 2025 or 2024 or 2023 or 2022 or 2021 or 2020 (Publication Years)
**WOS**
"menstrual cycle" (Topic) AND estrogen OR progesterone OR "follicular phase" OR "luteal phase" OR menstruation OR ovulation (Topic) AND injury (Topic) and Review Article (Exclude – Document Types) and English or Spanish (Languages) and 2026 or 2025 or 2024 or 2023 or 2022 or 2021 or 2020 (Publication Years)
**Cochrane Library**
("menstrual cycle" OR estrogen OR progesterone OR "follicular phase" OR "luteal phase" OR menstruation OR ovulation) AND (injury)
