# Supplementary material for: The Influence of the Phases of the Menstrual Cycle on Intrinsic Injury Risk Factors in Eumenorrheic Female Athletes or Physically Active Women—A Systematic Review
Source: Sports (Basel). 2026 Jul 10;14(7):297. doi: 10.3390/sports14070297 (PMC13416997; doi:10.3390/sports14070297)
Supplement: Supplementary file 1 [file sports-14-00297-s001.zip › sports-4372148-Table S2.pdf]

Supplementary Table S2.

Studies excluded after full text reading with the reason for exclusion.

	Reason for exclusion
**Ameer et al. (2025) [1]**	Less than three menstrual cycle phases analyzed
**Arenas-Pareja et al. (2026) [2]**	No intrinsicc injury risk factor
**Arslan and Ercan (2025) [3]**	No intrinsicc injury risk factor
**Baghban Baghdadabad & Mohaghegh (2024) [4]**	Less than three menstrual cycle phases analyzed
**Barbosa et al. (2023) [5]**	No intrinsicc injury risk factor
**Barlow et al. (2024) [6]**	No intrinsicc injury risk factor
**Baumgartner et al. (2023) [7]**	No intrinsicc injury risk factor
**Bouzigues et al. (2026) [8]**	No intrinsicc injury risk factor
**Cheng et al. (2021) [9]**	No intrinsicc injury risk factor
**Contarli & Ozmen (2024) [10]**	No correct intrinsic injury risk factor
**Darragi et al. (2024) [11]**	Less than three menstrual cycle phases analyzed
**Dehghan et al. (2024) [12]**	Less than three menstrual cycle phases analyzed
**Everaert et al. (2024) [13]**	Less than three menstrual cycle phases analyzed
**Ferrer et al. (2025) [14]**	No intrinsicc injury risk factor
**Ghomi et al. (2023) [15]**	Wrong population
**Gilmer et al. (2020) [16]**	Less than three menstrual cycle phases analyzed
**Gilmer & Oliver (2020) [17]**	Less than three menstrual cycle phases analyzed
**Guisado-Cuadrado et al. (2024) [18]**	No correct intriniec injury risk factor
**Ham et al. (2020) [19]**	Less than three menstrual cycle phases analyzed
**Hu et al. (2020) [20]**	Wrong population
**Ikarashi et al. (2020) [21]**	No correct intrinsic injury risk factor
**Iwanska et al. (2021) [22]**	Less than three menstrual cycle phases analyzed
**Johnson & Shields (2024) [23]**	No correct intrinsic injury risk factor

**Khowailed et al. (2022) [24]**	Less than three menstrual cycle phases analyzed
**Khowailed & Lee (2021) [25]**	Less than three menstrual cycle phases analyzed
**Kirschbaum et al. (2026) [26]**	No intrinsicc injury risk factor
**Krishnan et al. (2025) [27]**	Less than three menstrual cycle phases analyzed
**Lago-Fuentes et al. (2021) [28]**	No intrinsicc injury risk factor
**Legerlotz & Nobis (2022) [29]**	Incorrect study design
**Marshall & Chimea (2024) [30]**	Incorrect study design
**Martin et al. (2021) [31]**	No intrinsec injury risk factor
**Maruyama et al. (2021) [32]**	Wrong population
**McGrath et al. (2025) [33]**	No correct intrinsic injury risk factor
**Mizuta et al. (2025) [34]**	Wrong population
**Morales et al. (2023) [35]**	No correct intrinsic injury risk factor
**Oleka et al. (2020) [36]**	Incorrect study design
**Peinado et al. (2021) [37]**	Incorrect study design
**Pessali-Marques et al. (2024) [38]**	No correct intrinsic injury risk factor
**Puranda et al. (2023) [39]**	Incorrect study design
**Raj et al. (2023) [40]**	Incorrect study design
**]Reyes et al. (2023) [41]**	Wrong population
**Ronca et al. (2025) [42]**	Wrong population
**Roditi et al. (2024) [43]**	Less than three menstrual cycle phases analyzed
**Rodríguez et al. (2024) [44]**	Less than three menstrual cycle phases analyzed
**Santos et al. (2025) [45]**	No intrinsicc injury risk factor
**Shagawa et al. (2021) [46]**	Wrong population
**Sharma et al. (2024) [47]**	Less than three menstrual cycle phases analyzed
**Sommerfield et al. (2020) [48]**	Wrong population
**Suzuki-Yamanaka et al. (2025) [49]**	No intrinsicc injury risk factor
**Tagawa et al. (2024) [50]**	Less than three menstrual cycle phases analyzed
**Taş & Aktaş (2020) [51]**	Wrong population
**Van Rosen et al. (2020) [52]**	No intrinsicc injury risk factor
**Vico-Moreno et al. (2022) [53]**	Less than three menstrual cycle phases analyzed

**Witkos et al. (2022) [54]**	No intrinsicc injury risk factor
**Wojnowich & Dhani (2022) [55]**	Incorrect study design

Supplementary references:

1. Ameer, M.A.; Al Abbad, A.M.; Alsalem, J.O.; Alkhaldi, N.M.; Alanazi, K.O.; Alkawikbi, S.F.; Alhadi, M.F.; Alasker, S.A.; Alsirhani, H.S.; Alanazi, F.; et al. Effect of early follicular phase of menstrual cycle on hip flexion-extension isokinetic parameters in sedentary female students. *Heal. Care Women Int.* **2025**, *46*, 966–981, https://doi.org/10.1080/07399332.2024.2445239.
2. Arenas-Pareja, M.d.L.Á.; Lopez-Sierra, P.; Ibáñez, S.J.; García-Rubio, J. Study of menstrual cycle in training load variability in female basketball players. *J. Hum. Sport Exerc.* **2025**, *21*, 138–153, https://doi.org/10.55860/g0y2ke12.
3. Arslan, E.; Ercan, S. The effect of menstrual cycle phase changes on error scores in Sportive Movements in female athletes. *Physician Sportsmed.* **2025**, https://doi.org/10.1080/00913847.2025.2477977.
4. Baghdadabad, M.B.; Mohaghegh, S. Balance test results in different hormonal statuses of the menstruation cycle. Are females more susceptible to lower extremities injuries on different days of their menstruation cycles?. *Phys. Ther. Sport* **2023**, *65*, 54–58, https://doi.org/10.1016/j.ptsp.2023.11.003.
5. Barbosa, D.; De Souza Vidal, T.M.; Do Nascimento, G.R.; Costa, K.B.; Brito, A. de F.; Costa, M. da C.; Ribeiro de Lima, R.C.; Fernandes da Costa, M.S.; Dos Santos, W.R. Analysis of Injury Incidence in Athletes throughout the Phases of the Menstrual Cycle. Journal of Physical Education & Sport 2025, 25, 877. doi:10.7752/jpes.2025.04095
6. Barlow, A.; Blodgett, J.M.; Williams, S.; Pedlar, C.R.; Bruinvels, G. Injury Incidence, Severity, and Type Across the Menstrual Cycle in Female Footballers: A Prospective Three Season Cohort Study. *Med. Sci. Sports Exerc.* **2024**, *56*, 1151–1158, https://doi.org/10.1249/mss.0000000000003391.
7. Baumgartner, S.; Bitterlich, N.; Geboltsberger, S.; Neuenschwander, M.; Matter, S.; Stute, P. Contraception, female cycle disorders and injuries in Swiss female elite athletes—a cross sectional study. *Front. Physiol.* **2023**, *14*, 1232656, https://doi.org/10.3389/fphys.2023.1232656.
8. Bouzigues, T.; Candau, R.; Äyrämö, S.; Maurelli, O.; Prioux, J. Injury risk and workload analysis in elite adolescent female volleyball players using machine learning. *J. Sports Med. Phys. Fit.* **2026**, *66*, 72–81, https://doi.org/10.23736/s0022-4707.25.16744-3.
9. Cheng, J.; Santiago, K.A.; Abutalib, Z.; Temme, K.E.; Hulme, A.; Goolsby, M.A.; Esopenko, C.L.; Casey, E.K. Menstrual Irregularity, Hormonal Contraceptive Use, and Bone Stress Injuries in Collegiate Female Athletes in the United States. *PM&R* **2021**, *13*, 1207–1215, https://doi.org/10.1002/pmrj.12539.
10. Contarli, N.; Ozmen, T. Effect of the menstrual cycle on knee joint position sense and dynamic balance. *J. Bodyw. Mov. Ther.* **2024**, *40*, 1791–1794, https://doi.org/10.1016/j.jbmt.2024.10.030.

11. Darragi, M.; Zouhal, H.; Bousselmi, M.; Karamti, H.M.; Clark, C.C.T.; Laher, I.; Hackney, A.C.; Granacher, U.; Zouita, A.B.M. Effects of In-Season Strength Training on Physical Fitness and Injury Prevention in North African Elite Young Female Soccer Players. *Sports Med. - Open* **2024**, *10*, 1–20, https://doi.org/10.1186/s40798-024-00762-0.
12. Dehghan, F.; Soori, R.; Yusof, A. Mudanças na flacidez do joelho com esteroides sexuais durante as fases do ciclo menstrual em mulheres atletas e não atletas. *Rev. Bras. de Ortop.* **2024**, *59*, e29–e37, https://doi.org/10.1055/s-0043-1771007.
13. Everaert, K.; Fierens, B.; Steinberg, N.; Balagué, F.; Gielen, P.; Gielen, J.; van Breda, E.; Roussel, N. Association Between Bone Maturation, Menstrual Cycle, Hypermobility, Scoliosis, and Low Back Pain in Pre-Professional Female Adolescent Dancers. *Med Probl. Perform. Artist.* **2024**, *39*, 162–168, https://doi.org/10.21091/mppa.2024.04019.
14. Ferrer, E.; Keay, N.; Balagué-Dobón, L.; Cáceres, A.; Jarrin, P.; Rodas, G.; González, J. Menstruation and injury occurrence; a four season observational study in elite female football players. *Front. Sports Act. Living* **2025**, *7*, 1665482, https://doi.org/10.3389/fspor.2025.1665482.
15. Ghomi, M.M.; Shariati, M.; Mokhtari, M.; Talebian, S.; Nowrozani, F.R. Investigation of the Effects of Estrogen and Progesterone Hormones on Active Knee Joint Position Sense in Healthy Women in Different Phases of a Menstrual Cycle. *Braz. Arch. Biol. Technol.* **2023**, *66*, e23220156, https://doi.org/10.1590/1678-4324-2023220156.
16. Gilmer, G.G.; Roberts, M.D.; Oliver, G.D. The Relationship between Serum Relaxin Concentrations and Knee Valgus. *Int. J. Sports Med.* **2020**, *41*, 182–188, https://doi.org/10.1055/a-1062-6664.
17. Gilmer, G.; Oliver, G.D. Preliminary Evaluation of Knee Kinetics in Female Athletes on Hormonal Contraceptives. *Int. J. Sports Med.* **2019**, *41*, 113–118, https://doi.org/10.1055/a-1034-7901.
18. Guisado-Cuadrado, I.; Romero-Parra, N.; Elliott-Sale, K.J.; Sale, C.; Díaz, Á.E.; Peinado, A.B. Influence of Menstrual Cycle and Oral Contraceptive Phases on Bone (re)modelling Markers in Response to Interval Running. *Calcif. Tissue Int.* **2024**, *115*, 382–392, https://doi.org/10.1007/s00223-024-01259-4.
19. Ham, S.; Kim, S.; Choi, H.; Lee, Y.; Lee, H. Greater Muscle Stiffness during Contraction at Menstruation as Measured by Shear-Wave Elastography. *Tohoku J. Exp. Med.* **2020**, *250*, 207–213, https://doi.org/10.1620/tjem.250.207.
20. Hu, X.; Li, J.; Wang, L. Sex Differences in Lower Limb Proprioception and Mechanical Function Among Healthy Adults. *Mot. Control.* **2020**, *24*, 571–587, https://doi.org/10.1123/mc.2020-0015.
21. Ikarashi, K.; Iguchi, K.; Yamazaki, Y.; Yamashiro, K.; Baba, Y.; Sato, D. Influence of Menstrual Cycle Phases on Neural Excitability in the Primary Somatosensory Cortex and Ankle Joint Position Sense. *Women's Heal. Rep.* **2020**, *1*, 167–178, https://doi.org/10.1089/whr.2020.0061.
22. Iwańska, D.; Kęska, A.; Dadura, E.; Wójcik, A.; Mastalerz, A.; Urbanik, C. The effect of the menstrual cycle on collagen metabolism, growth hormones

and strength in young physically active women. *Biol. Sport* **2021**, *38*, 721–728, https://doi.org/10.5114/biolsport.2021.107314.
23. Johnson, K.A.; Shields, R.K. Influence of the Menstrual Cycle and Training on the Performance of a Perturbed Single-Leg Squatting Task in Female Collegiate Athletes. *Orthop. J. Sports Med.* **2024**, *12*, https://doi.org/10.1177/23259671241251720.
24. Khowailed, I.A.; Lee, Y.; Lee, H. Assessing the differences in muscle stiffness measured with shear wave elastography and myotonometer during the menstrual cycle in young women. *Clin. Physiol. Funct. Imaging* **2022**, *42*, 320–326, https://doi.org/10.1111/cpf.12763.
25. Khowailed, I.A.; Lee, H. Neuromuscular Control of Ankle-stabilizing Muscles-specific Effects of Sex and Menstrual Cycle. *Int. J. Sports Med.* **2020**, *42*, 270–276, https://doi.org/10.1055/a-1236-3654.
26. Kirschbaum, E.M.; Henke, J.; Heyde, K.; Legerlotz, K. Hormonal Contraception, Menstrual Cycle Characteristics, and Lower Limb Injuries in Elite Female Team Sports—Identifying Factors Associated With Increased Injury Prevalence: A Cross-Sectional Study. *Heal. Sci. Rep.* **2026**, *9*, https://doi.org/10.1002/hsr2.71812.
27. Krishnan, K.; Jamali, M.N.Z.M.; Yoong, L.P.; Krishnan, P. The Effect of Menstrual Cycle Phases on Agility and Shoulder Endurance in Recreational Basketball Players. *Orthop. J. Sports Med.* **2025**, *13*, https://doi.org/10.1177/23259671251334606.
28. Lago-Fuentes, C.; Padrón-Cabo, A.; Fernández-Villarino, M.; Mecías-Calvo, M.; Muñoz-Pérez, I.; García-Pinillos, F.; Rey, E. Follicular phase of menstrual cycle is related to higher tendency to suffer from severe injuries among elite female futsal players. *Phys. Ther. Sport* **2021**, *52*, 90–96, https://doi.org/10.1016/j.ptsp.2021.08.008.
29. Legerlotz, K.; Nobis, T. Insights in the Effect of Fluctuating Female Hormones on Injury Risk—Challenge and Chance. *Front. Physiol.* **2022**, *13*, 827726, https://doi.org/10.3389/fphys.2022.827726.
30. Marshall, K.A.; Chimera, N.J. Reaction Time Across the Menstrual Cycle: A Critically Appraised Topic. *Int. J. Athl. Ther. Train.* **2024**, *29*, 307–315, https://doi.org/10.1123/ijatt.2023-0070.
31. Martin, D.; Timmins, K.; Cowie, C.; Alty, J.; Mehta, R.; Tang, A.; Varley, I. Injury Incidence Across the Menstrual Cycle in International Footballers. *Front. Sports Act. Living* **2021**, *3*, https://doi.org/10.3389/fspor.2021.616999.
32. Maruyama, S.; Yamazaki, T.; Sato, Y.; Suzuki, Y.; Shimizu, S.; Ikezu, M.; Kaneko, F.; Matsuzawa, K.; Hirabayashi, R.; Edama, M. Relationship Between Anterior Knee Laxity and General Joint Laxity During the Menstrual Cycle. *Orthop. J. Sports Med.* **2021**, *9*, https://doi.org/10.1177/2325967121993045.
33. McGrath, M.D.; Brown, N.A.; Radcliffe, C.R.; McKay, A.K.; Bull, J.; Harris, R.; Minahan, C.; Ackerman, K.E.; Burke, L.M.; Coltman, C.E. Do fluctuations in oestradiol and progesterone across the menstrual cycle affect mechanical stiffness in female athletes?. *J. Sci. Med. Sport* **2025**, https://doi.org/10.1016/j.jsams.2025.06.010.

34. Mizuta, R.; Maeda, N.; Tashiro, T.; Kawai, M.; Oda, S.; Ishida, A.; Yoshiara, R.; Arima, S.; Urabe, Y. Reproducibility of dynamic balance and anterior tibiofibular gap measurements in men and women: A menstrual cycle-based longitudinal study. *J. Orthop. Sci.* **2024**, *30*, 844–849, https://doi.org/10.1016/j.jos.2024.11.008.
35. Morales, M.; Balzan, T.; Da Silva, A.; Muller, C.; Pinheiro, E.; Ferreira, G. The menstrual cycle affects the perception of fatigue in futsal athletes. *Sci. Sports* **2023**, *38*, 741–745, https://doi.org/10.1016/j.scispo.2022.11.007.
36. Oleka, C.T. Use of the Menstrual Cycle to Enhance Female Sports Performance and Decrease Sports-Related Injury. *J. Pediatr. Adolesc. Gynecol.* **2020**, *33*, 110–111, https://doi.org/10.1016/j.jpag.2019.10.002.
37. Peinado, A.B.; Alfaro-Magallanes, V.M.; Romero-Parra, N.; Barba-Moreno, L.; Rael, B.; Maestre-Cascales, C.; Rojo-Tirado, M.A.; Castro, E.A.; Benito, P.J.; Ortega-Santos, C.P.; et al. Methodological Approach of the Iron and Muscular Damage: Female Metabolism and Menstrual Cycle during Exercise Project (IronFEMME Study). *Int. J. Environ. Res. Public Heal.* **2021**, *18*, 735, https://doi.org/10.3390/ijerph18020735.
38. Pessali-Marques, B.; Burden, A.M.; Morse, C.I.; Onambélé-Pearson, G.L. Musculoskeletal Morphology and Joint Flexibility-Associated Functional Characteristics across Three Time Points during the Menstrual Cycle in Female Contemporary Dancers. *J. Funct. Morphol. Kinesiol.* **2024**, *9*, 38, https://doi.org/10.3390/jfmk9010038.
39. Puranda, J.L.; da Silva, D.F.; Edwards, C.M.; Nagpal, T.S.; Souza, S.S.; Semeniuk, K.; Adamo, K.B. Association Between Reproductive Health Factors and Musculoskeletal Injuries in Female Canadian Armed Forces Members. *J. Women's Heal.* **2023**, *32*, 199–207, https://doi.org/10.1089/jwh.2021.0647.
40. Raj, R.D.; Fontalis, A.; Grandhi, T.S.P.; Kim, W.J.; Gabr, A.; Haddad, F.S. The impact of the menstrual cycle on orthopaedic sports injuries in female athletes. *Bone Jt. J.* **2023**, *105-B*, 723–728, https://doi.org/10.1302/0301-620x.105b7.bjj-2022-1262.r2.
41. Reyes, S.S.; Gómez, J.S.; Ponce, I.G.; Moraleda, B.R. Estudio descriptivo de las lesiones de ligamento cruzado en el fútbol femenino (Descriptive study of cruciate ligament injuries in female soccer). *Retos* **2023**, *50*, 172–179, https://doi.org/10.47197/retos.v50.96622.
42. Ronca, F.; Blodgett, J.; Bruinvels, G.; Lowery, M.; Raviraj, M.; Sandhar, G.; Symeonides, N.; Jones, C.; Loosemore, M.; Burgess, P. Attentional, anticipatory and spatial cognition fluctuate throughout the menstrual cycle: Potential implications for female sport. *Neuropsychologia* **2024**, *206*, 108909, https://doi.org/10.1016/j.neuropsychologia.2024.108909.
43. Roditi, E.-E.; Tsatalas, T.; Sakkas, G.K.; Koutedakis, Y.; Giakas, G.; Karatzaferi, C. Effects of Muscular Fatigue on Position Sense in Two Phases of the Menstrual Cycle. *J. Funct. Morphol. Kinesiol.* **2024**, *9*, 115, https://doi.org/10.3390/jfmk9030115.
44. Rodriguez, L.A.; Liu, Y.; Soedirdjo, S.D.H.; Thakur, B.; Dhaher, Y.Y. Oral Contraception Use and Musculotendinous Injury in Young Female Patients: A Database Study. *Med. Sci. Sports Exerc.* **2024**, *56*, 511–519, https://doi.org/10.1249/mss.0000000000003334.

45. Santos, W.R.; Júnior, F.C.; Barbosa, D.; Ginnari, K.V.; Dos Santos, A.K.S.G.; Timoteo, B.d.O.; Costa, M.d.C.; da Costa, M.S.F.; Paes, P.P. Injuries in Female Soccer Athletes: Risk of Injury Across Different Phases of the Menstrual Cycle. *Res. Q. Exerc. Sport* **2025**, *96*, 819–826, https://doi.org/10.1080/02701367.2025.2515968.
46. Shagawa, M.; Maruyama, S.; Sekine, C.; Yokota, H.; Hirabayashi, R.; Hirata, A.; Yokoyama, M.; Edama, M. Comparison of anterior knee laxity, stiffness, genu recurvatum, and general joint laxity in the late follicular phase and the ovulatory phase of the menstrual cycle. *BMC Musculoskelet. Disord.* **2021**, *22*, 1–7, https://doi.org/10.1186/s12891-021-04767-8.
47. Sharma, G.; Kumari, N.; Rai, R.H.; Kalra, S.; Bhatia, K.; Saher, T. Relationship between Functional Capacity, Fatiguability, Anxiety Level, Static, and Dynamic Balance in Young Adult Females During Follicular and Luteal Phase of Menstrual Cycle. *Med J. Dr. D.Y. Patil Vidyapeeth* **2024**, *17*, S301–S307, https://doi.org/10.4103/mjdrdypu.mjdrdypu_680_23.
48. Sommerfield, L.M.; Harrison, C.B.; Whatman, C.S.; Maulder, P.S. A prospective study of sport injuries in youth females. *Phys. Ther. Sport* **2020**, *44*, 24–32, https://doi.org/10.1016/j.ptsp.2020.04.005.
49. Suzuki-Yamanaka, M.; Hashiba, S.; Takahashi, R.; Sakamaki-Sunaga, M.; Iizawa, H.; Mamizuka, N. Incidence of Sports Injuries across Menstrual Phases in Eumenorrheic and Abnormal Cycles in Japanese Female Athletes: A Prospective Cohort Study. *Med. Sci. Sports Exerc.* **2025**, *57*, 1395–1401, https://doi.org/10.1249/mss.0000000000003679.
50. Tagawa, N.; Okamura, K.; Araki, D.; Sugahara, A.; Kanai, S. Influence of the menstrual cycle on static and dynamic kinematics of the foot medial longitudinal arch. *J. Orthop. Sci.* **2023**, *29*, 609–614, https://doi.org/10.1016/j.jos.2023.01.009.
51. Taş, S.; Aktaş, D. Menstrual Cycle does not Affect the Mechanical Properties of Muscle and Tendon. *Muscle Ligaments Tendons J.* **2020**, *10*, 11–16, https://doi.org/10.32098/mltj.01.2020.02.
52. von Rosen, P.; Heijne, A.; Frohm, A.; Fridén, C. Menstrual irregularity and use of oral contraceptives in female adolescent athletes in Swedish National Sports High Schools. *Int. J. Adolesc. Med. Heal.* **2017**, *32*, https://doi.org/10.1515/ijamh-2017-0113.
53. Vico-Moreno, E.; Sastre-Munar, A.; Fernández-Domínguez, J.C.; Romero-Franco, N. Motor Control and Regularity of Menstrual Cycle in Ankle and Knee Injuries of Female Basketball Players: A Cohort Study. *Int. J. Environ. Res. Public Heal.* **2022**, *19*, 14357, https://doi.org/10.3390/ijerph192114357.
54. Witkoś, J.; Błażejewski, G.; Hagner-Derengowska, M.; Makulec, K. The Impact of Competitive Swimming on Menstrual Cycle Disorders and Subsequent Sports Injuries as Related to the Female Athlete Triad and on Premenstrual Syndrome Symptoms. *Int. J. Environ. Res. Public Heal.* **2022**, *19*, 15854, https://doi.org/10.3390/ijerph192315854.
55. Wojnowich, K.; Dhani, R. Care of the Active Female. *Am. Fam. Physician* **2022**, *106*, 52–60.
